# Empowering Capabilities of People With Chronic Conditions Supported by Digital Health Technologies: Scoping Review

**DOI:** 10.2196/68458

**Published:** 2025-06-27

**Authors:** Messaline Fomo, Liyousew G Borga, Thomas Abel, Philip S Santangelo, Sara Riggare, Jochen Klucken, Ivana Paccoud

**Affiliations:** 1 Luxembourg Centre for Systems Biomedicine (LCSB) University of Luxembourg Esch-sur-Alzette Luxembourg; 2 Digital Medicine Group Department of Precision Medicine Luxembourg Institute of Health Strassen Luxembourg; 3 Institute of Social and Preventive Medicine (ISPM) University of Bern Bern Switzerland; 4 Department of Behavioural and Cognitive Sciences Faculty of Humanities, Education and Social Sciences University of Luxembourg Esch-sur-Alzette Luxembourg; 5 Participatory eHealth and Health Data Research Group Department of Women’s and Children’s Health Uppsala University Uppsala Sweden; 6 Centre Hospitalier de Luxembourg (CHL) Luxembourg Luxembourg

**Keywords:** patient empowerment, digital health technology, digital health, capability approach, chronic diseases

## Abstract

**Background:**

Patient empowerment is widely recognized for improving health outcomes, increasing patient satisfaction, and enhancing the overall effectiveness of health care. Digital health technologies (DHTs) contribute to this empowerment by keeping patients informed, involved, and engaged in their own health. However, more evidence is needed to better understand which aspects of empowerment patients value when using DHTs and how DHTs can support these values.

**Objective:**

Drawing on Sen’s capability approach, this paper conceptualizes patient empowerment in digital health by defining distinct capabilities, resources, and conversion factors that contribute to patient empowerment through DHTs.

**Methods:**

We based our scoping review on the methodology recommended by the Joanna Briggs Institute Manual for evidence synthesis and an a priori registered protocol. Papers were included if they focused on patient empowerment in relation to DHTs among patients with chronic diseases (cardiovascular diseases, diabetes, cancer, chronic respiratory diseases, and neurodegenerative diseases), with particular emphasis on the patient perspective. PubMed, Scopus, and Web of Science were searched for evidence published from January 2013 to April 2024. Data were extracted and thematically analyzed via a multidisciplinary workshop to identify empowerment components relevant to the capability framework, such as capabilities, DHTs as resources, and conversion factors.

**Results:**

Our analysis identified 3 core capabilities to achieve patient empowerment supported by DHTs: health information and knowledge management, self-management, and emotional and social support. DHTs as resources supported these capabilities through distinct functional components, including informing patients, communication with the health care team, monitoring, behavior change interventions, individualized feedback, or peer support, each contributing to a varying degree. Conversion factors such as demographic and socioeconomic status, digital literacy, disease status, perceived value of DHTs, sociocultural values and norms, doctor-patient relationship, connectivity, and cost influenced the development of empowering capabilities resulting from using DHTs.

**Conclusions:**

While the capabilities related to patient empowerment in DHTs were clearly distinguishable, our analysis revealed a notable interconnectedness among these components. Our conceptualization of patient empowerment serves as a valuable resource for researchers seeking to understand or assess patient empowerment via DHTs. It also provides guidance for DHT developers, helping them design DHTs that enhance valued capabilities and account for the conversion factors and ultimately promote patient empowerment across diverse population groups.

## Introduction

With the rising burden of chronic diseases, health care systems worldwide are shifting toward patient-centered strategies to deliver high-quality, cost-effective care. A key concept in this regard is patient empowerment, defined as “a process that helps people gain control over their own lives and increases their capacity to act on issues they themselves define as important” [1]. The advantages of patient empowerment have been well-documented across various chronic diseases such as diabetes, cancer, cardiovascular disease, and heart failure [2,3]. Empowered patients tend to have a better understanding of their health, make informed decisions about their treatment, take greater responsibility for their care, and demonstrate better adherence to prescribed therapies [4,5]. However, despite the proven benefits and the growing emphasis for patients to become comanagers in their health care [6,7], substantial challenges persist in translating the concept of patient empowerment into practical, measurable strategies that can be effectively integrated into health care systems and enhanced through supportive services.

Digital health technologies (DHTs), specifically patient-facing DHTs as defined by the digital therapeutic alliance [8], have emerged as innovative tools to foster patient empowerment. These technologies are intended primarily for patient use, offering patient-facing features across the categories of health and wellness, patient monitoring, care support, digital diagnostics, and digital therapeutics. Features that make DHTs suitable for empowering patients are their capacity to provide education, enable remote access to health care providers, facilitate monitoring and support, and provide timely feedback [9]. While DHTs provide new opportunities to empower patients [10], there remains a lack of clear guidance on how to operationalize and apply this dynamic, multidimensional concept in the design and evaluation of DHTs. Even though established frameworks for studying empowerment exist [11-13], there remains a gap in frameworks specifically addressing empowerment within the digital health context. Furthermore, research suggests that patients can also be empowered outside the traditional patient-provider interaction [6,14], reinforcing the importance of understanding how DHTs contribute to this process in a range of situations beyond traditional clinical encounters.

However, much of the research is focused on 2 key areas: leveraging tools like clinical decision support systems [15] to make the health care system and its providers more effective and expand their services or developing methods to measure and monitor biomedical outcomes [16-18]. Additionally, studies have also documented the benefit of DHTs on health care service use and patient health outcomes, such as disease knowledge, patient engagement, treatment adherence, and self-management [19]. Although equally important, the emphasis on technology and the clinical aspect often overshadows the perspectives and needs of patients. This oversight can limit the ability of DHTs to provide direct value to the primary users—the patients themselves [20]. Even when research addresses these issues, it remains fragmented and fails to offer a clear understanding of how DHTs contribute to patient empowerment.

This fragmentation is evident in existing systematic reviews, which tend to focus narrowly on specific technologies (eg, wearable sensors, mobile apps, and patient portals) [21-23], particular functionalities (eg, digital therapeutics and patient monitoring) [24], individual diseases (diabetes or cardiovascular care) [25], or selected outcomes [26]. Some of the reviews are now obsolete in this rapidly changing technological landscape [9], while others emphasize on the design features of patient-facing DHTs [27] or identify barriers and facilitators to their adoption [24,28]. These gaps, along with the growing promise that DHTs will empower patients, mean we need to understand how empowerment occurs in the digital health context from the patient’s perspective and how their unique features influence this process.

Given the complex nature of empowerment and how it can be supported by DHTs, the use of a theoretical framework can be valuable. The capability approach introduced by Amartya Sen and further developed in collaboration with Nussbaum can be useful in this regard [29]. Originally developed in the field of economics, the capability approach has gained traction in health care to conceptualize multidimensional constructs and evaluate health and social care interventions [30]. Recognizing that individuals differ in the way they transform resources into meaningful outcomes (functioning), Sen proposed assessing people’s capabilities, that is, the real opportunities people have to achieve valuable outcomes related to their health and well-being, such as staying healthy or feeling empowered. Having different capabilities expands patients’ agency, enabling them to act on matters that are important to their health and well-being, regardless of the consequences of that action. Nussbaum [31], for instance, identified 10 central capabilities, which represent real opportunities that could be actualized based on an individual’s personal, social, and environmental circumstances, called conversion factors. Some examples related to health and health care include being able to (1) live a normal life span; (2) have good bodily health; (3) experience bodily integrity; (4) use one’s senses, imagination, and thought; (5) experience emotions and form attachments; (6) exercise practical reason; and (7) control one’s environment. Nussbaum further stressed the flexibility of the framework and the need to adapt the definition of these capabilities to the specific resource being studied—in our case, patient-facing DHTs.

**Figure 1:**
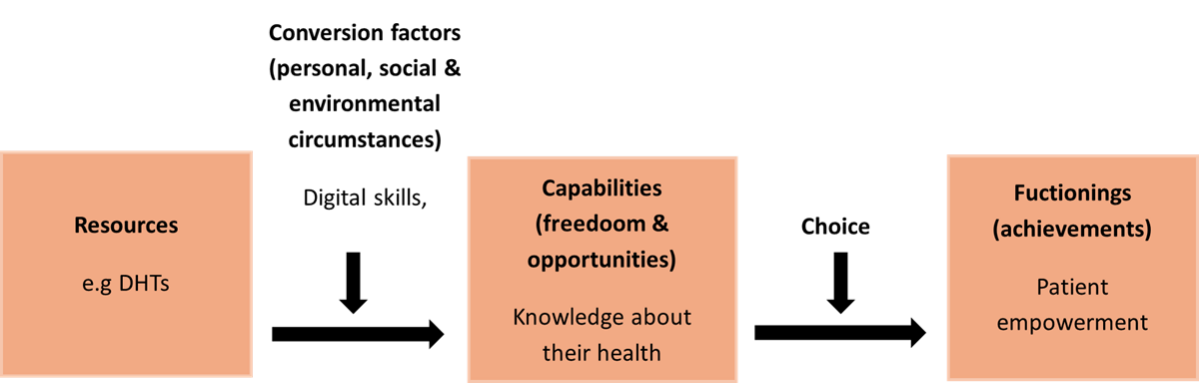
Relationship between core components of the capability approach applied to patient empowerment in the digital health context. DHT: digital health technology.

Applying the capability framework to our context, we argue that DHTs serve as distinct resources that are designed to facilitate patient empowerment, which is a desired outcome (functioning). However, the extent to which patients can achieve empowerment depends on their capabilities, shaped by their personal, social, and environmental circumstances such as their digital skills and internet access. For example, if a patient’s ability to learn more about their health condition is a valued capability for their empowerment, then they need to be able to use DHTs for this purpose, which requires sufficient digital literacy (a personal conversion factor) and internet access (an external conversion factor). Figure 1 illustrates the core components of the capability approach applied to patient empowerment through DHTs based on adapted concepts from Robeyns [29].

Therefore, this study aims to use the capability approach to better understand and conceptualize the process of patient empowerment in relation to DHTs and their functional components by synthesizing available evidence in a systematic way. The main objectives are (1) to identify empowering capabilities that are valued by people with chronic conditions when using DHTs, (2) to identify and map DHTs as a resource in terms of DHTs and their functional components that enhance specific empowering capabilities, and (3) to identify conversion factors that influence the achievement of empowering capabilities when using DHTs.

Addressing these objectives is essential, as it offers a patient-centered framework for understanding patient empowerment and its process. This could serve as a guide for DHT developers, researchers, and other stakeholders when designing and evaluating DHTs that enhance and support the empowering capabilities.

## Methods

### Study Design

We conducted a scoping review to explore and synthesize evidence on contemporary DHTs across various chronic diseases and to identify concepts of patient empowerment in relation to DHTs. The methodology of the scoping review was guided by the Joanna Briggs Institute scoping review framework [32] and followed the PRISMA-ScR (Preferred Reporting Items for Systematic Reviews and Meta-Analyses extension for Scoping Reviews) for reporting (Multimedia Appendix 1) [33]. A protocol was developed and registered on the Open Science Framework prior to commencing the review that outlined the intended approach and method [34], which are summarized below.

### Eligibility Criteria

The population, concept, and context framework was used to ensure the comprehensive inclusion of relevant papers and the exclusion of irrelevant ones (Textbox 1).

**Inclusion and exclusion criteria based on the population, concept, and context framework.**

**Inclusion criteria**

- Population: Adults aged ≥18 years with chronic diseases mainly cardiovascular diseases, diabetes mellitus, chronic respiratory diseases, cancer, and neurodegenerative diseases.
- Concept: Investigate patient empowerment as a main concept in the context of digital health technologies (DHTs), focusing on the patient’s perspective; focus on patient-facing DHTs.
- Context: Investigated patient empowerment from the perspective of patients and within the health care setting.
- Language: Papers written in English.

**Exclusion criteria**

- Population: Focus on young patients or adult patients without chronic diseases.
- Concept: Investigate patient empowerment outside the context of DHTs; DHTs used to improve the health care system or those that support only the exchange of information without any aspect of patient interaction or behavior such as SMS text messaging, emails, and online health communities.
- Context: Investigated patient empowerment outside the health care context or not focusing on patient perspective.
- Language: Other languages.

Within each chronic disease group, we focused on the most prevalent ones: cardiovascular diseases (hypertension, coronary artery disease, and heart failure), diabetes mellitus, cancer (lungs, breast, prostate, colon, and rectum), chronic respiratory diseases (asthma and chronic obstructive pulmonary disease), and neurodegenerative diseases (Parkinson and Alzheimer disease). Although representing a heterogeneous patient group, these conditions share similar expectations that patient empowerment will enable them to effectively manage their condition over time [6,7].

Because of the diverse landscape of DHTs, we focused on patient-facing DHTs, as defined by the digital therapeutic alliance [8]. We acknowledge the potential overlap of this classification with features targeting physicians, payers, or health systems. However, this review specifically focuses on technologies with patient-facing features. We further excluded technologies that only supported information exchange without any aspect of patient interaction or behavior such as SMS text messaging, emails, and online health communities.

### Search Strategy

Database searches included PubMed, Scopus, and Web of Science between January 2013 and December 2023 to capture more recent technologies. We selected these databases for their comprehensive, interdisciplinary coverage of health care and digital health literature relevant to our research on the conceptualization of patient empowerment in the digital health context with limited redundancy. The search was later extended to April 8, 2024, to capture subsequently published papers. Gray literature was not searched.

The search strategy used a combination of search terms related to our underlying concepts (“patient empowerment” and DHTs), and these terms were adapted to fit the syntax of each database (Table S1 in Multimedia Appendix 2) [35-75]. Initially, a preliminary search was done on PubMed to identify relevant text words in the title and abstract as well as any index terms that could serve as alternate search terms. Subsequently, a more detailed search was conducted across all other databases. We manually searched the reference lists of all eligible studies to identify additional studies that met the inclusion criteria. The detailed search strategy used for PubMed, which is the basis for all other database searches, can be found in Table S1 in Multimedia Appendix 2.

The search terms did not include a specific term for chronic diseases due to the diversity of chronic conditions to maintain a broad scope and avoid restricting the search to predefined disease categories. Instead, the eligibility criteria were applied during the screening stage to identify studies relevant to the chronic disease of interest. This approach ensured that our search terms were manageable while minimizing the risk of unintentionally excluding relevant studies due to variations in disease terminology across databases and disciplines.

### Selection Process

The paper selection was conducted by the first 2 authors (MF and LB) in 2 phases. In the initial phase, MF checked titles and abstracts to determine the relevance of papers for retrieval of the full text. In the second phase, both MF and LB performed a full-text screening against the inclusion and exclusion criteria in Textbox 1, documenting reasons for exclusion. Any discrepancies between screeners were resolved through discussion and consensus.

### Data Extraction and Analysis

A predefined Microsoft Excel form was used to extract relevant information from each paper, including key findings and themes identified by the authors. The analysis aimed to highlight three main aspects: (1) resources in terms of the DHTs and their functional components identified from the description of technologies or digital health intervention in each study; (2) personal, social, and environmental conversion factors perceived by patients that hindered or supported their empowerment; and (3) capabilities related to empowerment inferred by patients in terms of values, enablement, or opportunities facilitated by DHTs. MF performed the data extraction and initial analysis, while IP verified the data extraction and analysis conducted.

Thematic analysis of the extracted data followed Braun and Clarke’s 6-step process [76]. MF initially coded text elements from each paper relating to capabilities based on its explicit meaning and content from the papers. The initial codes from all papers were then grouped together based on their similarities and differences captured in the meaning of the initial codes, forming potential subthemes. These subthemes represent emerging patterns in the data. Due to the persistent overlap between the subthemes, we further grouped them into broader overarching themes through a collaborative process with a multidisciplinary team. The team comprised medical doctors, psychologists, patient experts, social scientists, and health economists. They discussed, revised, and further grouped the initially developed subthemes into distinct, well-defined themes. Through a consensus, the team established the final thematic categories representing capabilities that grouped similar concepts together while ensuring that distinct ideas remained separate. Throughout this process, we relied on existing terminologies around patient empowerment from the patient perspective to develop our themes [13]. A deductive analytic approach was used to identify the functional components of DHTs, and the terminologies were adapted from the UK National Institute for Health and Care Excellence evidence standard framework [77] and digital therapeutic alliance classification framework [8].

## Results

### Characteristics of Eligible Studies

**Figure 2:**
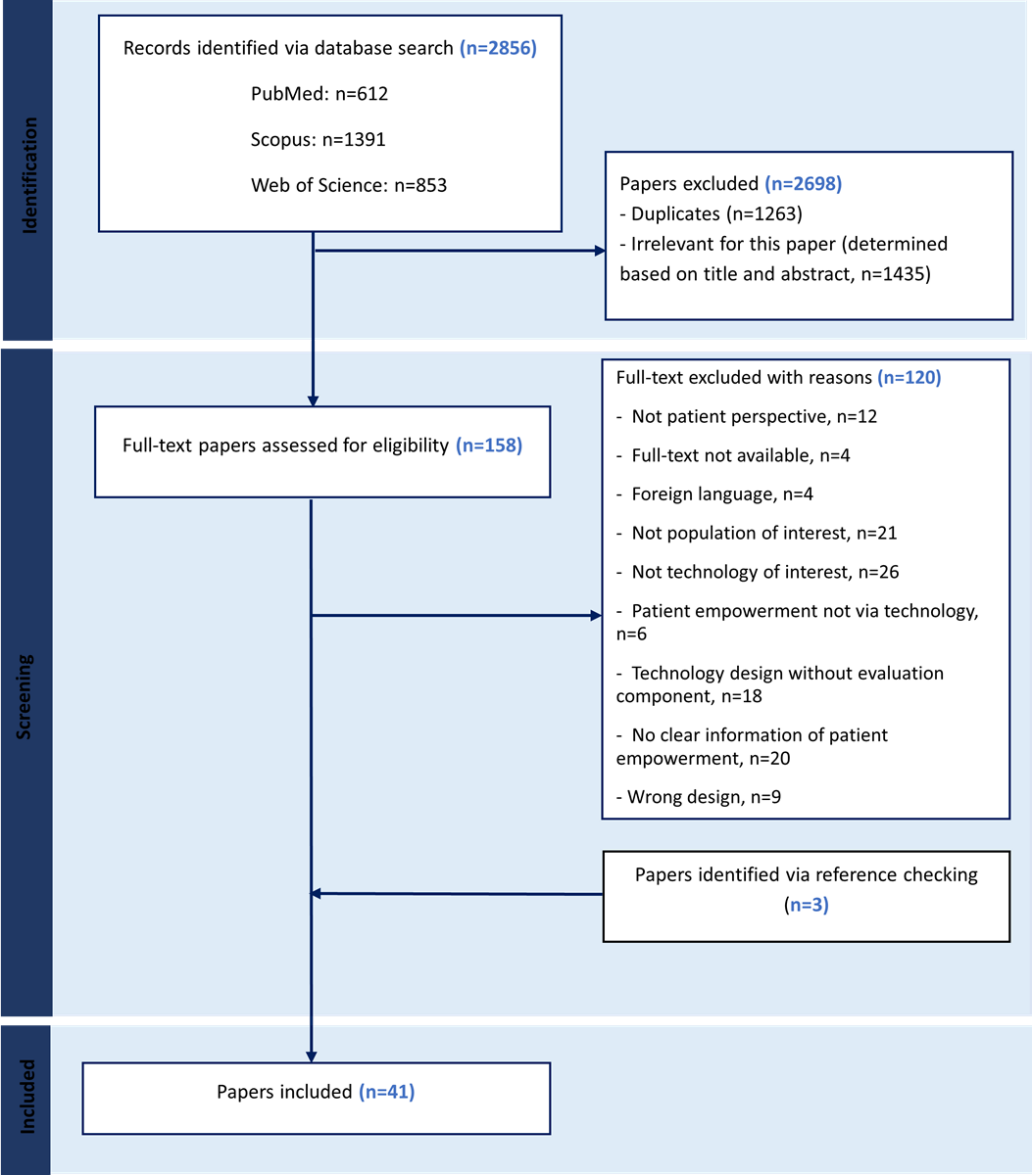
Flow diagram showing the screening process.

The initial search identified 2856 studies containing 1263 duplicates, which were removed, leaving 1593 papers for the title and abstract screening against the inclusion criteria. We retrieved 158 papers for full-text reviews, of which 38 were eligible for the final analysis, with an additional 3 added after reviewing their reference lists. Figure 2 outlines the steps in detail. In addition, a summary of the included studies can be seen in Table S2 in Multimedia Appendix 2.

More than half of the studies were conducted in Europe and North America (35/40, 80%), with a minority in Asia, Middle East, and Oceania (Table 1). Of the studies included, the majority focused on patients with diabetes [35-49], while the others were on patients with cancer [50-57], cardiovascular diseases [58-63,75], chronic obstructive pulmonary disease [64,65], and Parkinson disease [66,67], and the rest were a combination of 2 or more pathologies. Different types of DHTs were used, such as mobile apps [37-39,45,47,51,55,58-61,68,69,75], web-based interventions [35,36,53,56,57,62,70], telemonitoring devices [48,65,71], connected devices, for example, connected to other medical devices like a glucometer or blood pressure monitor that enables 2-way transmission of collected data [46,68], wearable sensors [66], and a combination of 2 or more technologies, for example, wearable sensor, mobile app, and patient portal [40-42,44,52,72].

**Table 1:** Descriptive characteristics of included studies

Study characteristics		Values, n (%)
**Country setting**		
	Europe	23 (57.5)
	North America	12 (30)
	Asia	2 (5)
	Oceania	2 (5)
	Middle East	1 (2.5)
**Disease population**		
	Diabetes	14 (34.2)
	Cardiovascular diseases	8 (19.5)
	Cancer	8 (19.5)
	Chronic respiratory diseases	3 (7.3)
	Neurodegenerative diseases	2 (4.9)
	Combination of diseases	6 (14.6)
**Type of DHT^a^ used**		
	Mobile- or computer-based apps	18 (43.9)
	Web-based interventions	8 (19.5)
	Telemonitoring system	5 (12.2)
	Connected devices	2 (4.9)
	Wearable device	1 (2.4)
	Software	1 (2.4)
	Combination of 2 or more technology	6 (14.6)
**Patient facing features**		
	Patient monitoring	26 (63.4)
	Health and wellness	7 (17.1)
	Care support	7 (17.1)
	Digital therapeutics	1 (2.4)
	Digital diagnostics	0 (0)

^a^DHT: digital health technology.

### Capabilities for Patient Empowerment Supported by Digital Health Technologies

#### Overview

**Figure 3:**
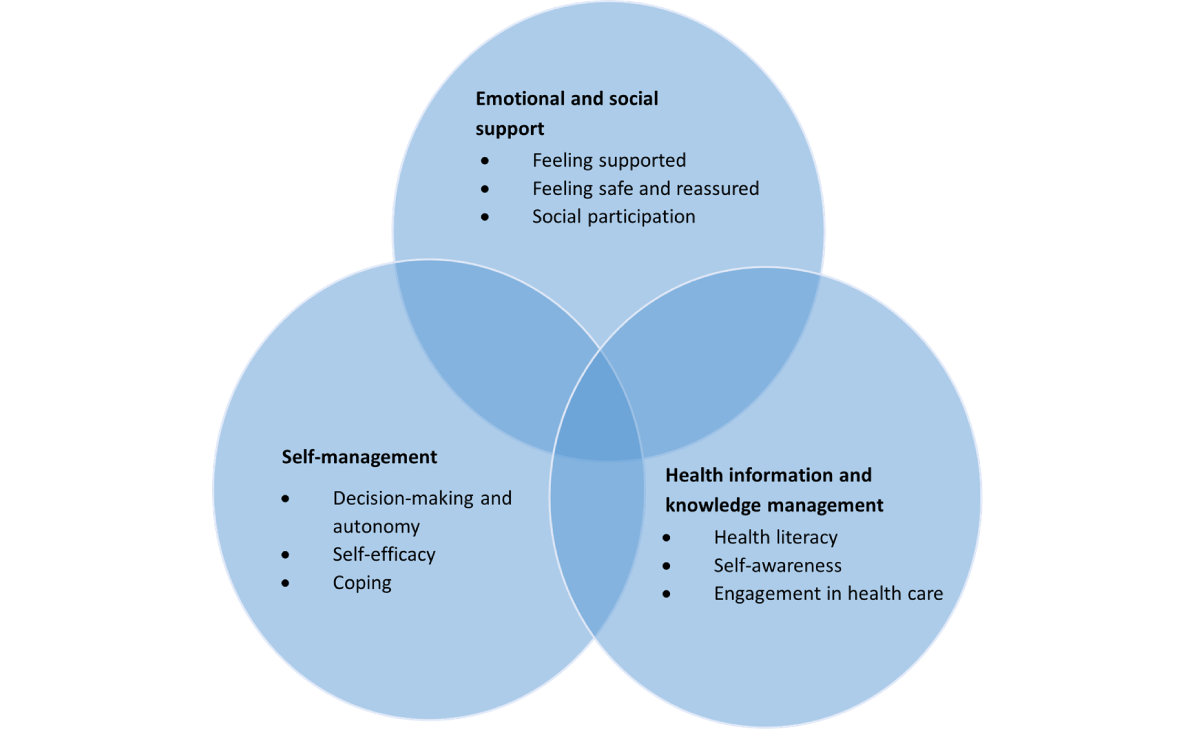
Capabilities for patient empowerment supported by digital health technologies.

Using Braun and Clarke’s 6-step process of thematic analysis [76], 3 core capabilities to achieve empowerment were identified (Figure 3): emotional and social support, self-management, and health information and knowledge management.

**Figure 4:**
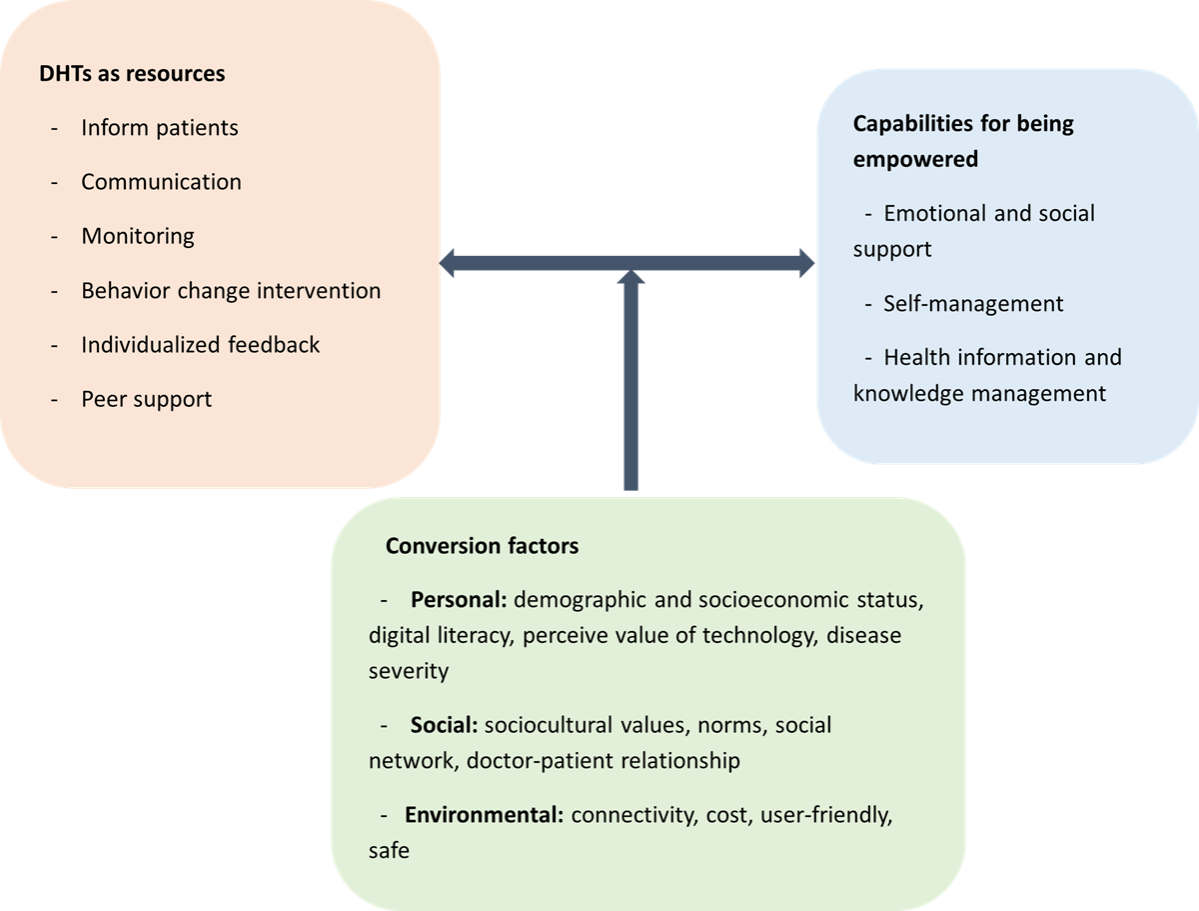
Relationship outlining the process by which DHTs lead to empowering capabilities through conversion factors. DHT: digital health technology.

The capabilities, functional components of DHTs, and conversion factors are presented in Figures 3 and 4 and described in detail below.

#### Emotional and Social Support

Patients valued DHTs, as they provided them with emotional and social support. This support was reflected in the feeling of safety patients had when using DHTs, the support obtained from their network, and their ability to participate in social life.

Patients reported feeling safe when using DHTs, as it enabled them to monitor symptom fluctuations and receive regular updates from their health care provider, leading to increased comfort, reduced stress, and reassurance [41,47,50,53,54,56,61,64,71]. Additionally, patients also valued DHTs, as they allowed them to regain their social life and pursue activities that mattered most to them, independent of their disease-management activities [41,50,62,70]. Studies also highlighted a connection between emotional and social support. For instance, the reassurance gained from symptom monitoring or being watched by their health care team enabled patients to connect with their support network outside of the hospital [36,40,48,53], offering a platform to share experiences and monitor their health [56,61,75]. Furthermore, many patients relied on symptom tracking through DHTs to make decisions about participating in household tasks or going for family outings [62,65]. Caregivers could use patients’ health data to offer support and reinforce healthy behaviors outside the clinical settings [47]. Patients also found satisfaction in sharing their own experience on disease management with peers and supporting caregivers in return [53].

#### Self-Management

This capability reflects the patient’s ability to make health-related decisions, develop cognitive and physical skills to manage their condition, a core aspect of self-efficacy, and adapt to the challenges of living with a chronic condition through coping strategies. Patient’s ability to make informed, autonomous decisions regarding their health allowed them to gain control of both their personal lives [63,73] and experiences with the illness [36,41,49,55,56,60,61,64,69]. This manifested as taking proactive steps to manage their disease [38,53,65], such as seeking extra medical consultations during periods of disease deterioration, limiting their daily activities to prevent worsening of symptoms [50,57,60,64], or engaging in self-management to improve their health [36-38,40,63,64]. Access to DHTs provided patients with confidence in assessing their symptoms and taking action [41,42,48-50,54,62,63,66]. It also motivated patients to improve their health and actively manage their condition [47,48,58,61,67]. Furthermore, DHTs provided patients with strategies for dealing with anxiety and stress [62] and daily life challenges [40,49] while also helping them cope with the emotional expectations and psychosocial aspects of the disease [53,70]. Other coping strategies developed were their ability to seek help when needed and to adopt a positive attitude toward their condition [56].

#### Health Information and Knowledge Management

This capability represents patients’ wish to gain knowledge about their health condition and use it for their empowerment and well-being. It encompasses health literacy, self-awareness, and engagement in health care. Patients used DHTs to review their medical records or communicate directly with their health care provider, which provided them with simplified information about their health [57,59]. Being aware of their own body [49,53], as well as their health status, symptoms, management strategies, or lifestyle changes [36,37,40,44,47,48,66,71], was also valued by patients. This awareness helped them better grasp their health condition and management recommendations [35,37,40,44,47,48,52,53,56,58,60-66,68,70,71]. With this knowledge, patients were able to engage in health care and improve their communication and interaction with both the health care team and the broader system [47,53,55,70]. For example, using DHTs to monitor symptoms enabled patients to prepare for clinic visits and to ask pertinent questions about their health [35,36]. Its use also enabled patients to receive timely feedback on their health status [47], exchange health-related information [51,71], and lead conversations with their health care providers during the consultation [60,61]. Through this, patients were able to maintain a collaborative partnership with health care providers, thereby strengthening the patient-provider relationship [47,53].

### DHTs as Resources

This review identified 6 functional components of DHTs as resources to achieve patient empowerment. These resources are informing patients, communication, monitoring, behavior change intervention, individualized feedback, and peer support (Table 2).

**Table 2:** Digital health technologies (DHTs) as resources and description.

DHTs as resources	Description
Informing patients	Providing information to patients to help them understand healthy living and illness [37,38,43,52-54,60,69,72]. This information could be in the form of text, videos, dialogue format with chat bot, or assignments and assessments and is accessible via mobile apps [47,55,59,70], website, or patient portal [36,57].
Communication	Allows for bidirectional communication between patients and the health care team where clinical advice or disease management support is provided by a health care professional [39,43,48,55,60,63-65]. This involves the use of instant messaging apps, phone calls, or web-based platforms.
Monitoring	Capturing data on health parameters (disease-related symptoms, health-related activities, and patient-reported outcomes) either automatically or by the patient and transmitting these data to the health care team to inform clinical management decision [35,38-40,43,47,48,50,54,60,63,65,66,69,71].
Behavior change intervention	Modifying patient behaviors related to health issues. It involves the use of health coaching strategies where patients are engaged in the creation of self-management activities, setting goals, and receiving guidance to achieve these goals [36,37,40,42,48,52,59,62,72]. These can be achieved through phone calls or self-training videos. In addition, by using motivational tools to prompt patients to complete tasks related to the management of disease and health care access, for example, reminders or notification prompts [41,43,53,65,69].
Individualized feedback	Uses patient-generated monitoring data to provide disease-related feedback [41,44,45,58,61,68,75] or recommendations on risk stratification [49], treatment [45,56], or lifestyle changes. This also includes visualization of monitoring data.
Peer support	Allows patients to get in touch with and gain support from other patients with similar conditions, for example, through web forums [36,37,51].

### Conversion Factors

The review further identified 3 broad categories of conversion factors—personal, social, and environmental—which may be either internal or external to the individual. These categories are summarized as follows.

#### Personal Factors

Patients’ sociodemographic factors significantly influence their capabilities and thus their potential for empowerment. Younger and more educated patients were more likely to achieve these capabilities [49,55], while patients with low socioeconomic status or a migrant background faced challenges due to language barriers that hindered their use of DHTs and thus their capabilities [68]. Digital literacy was also important [58,59], as it boosted their confidence and made them more proactive and engaged in care [58]. Furthermore, some patients experienced anxiety from information overload [68], and those with well-controlled symptoms did not find any value in using DHTs [49]. The perceived added value of the DHTs in terms of appropriateness of the content, recommendations, and user experience and satisfaction fostered patient empowerment [55,58,59,74].

#### Social Factors

Cultural norms and values influence the development of capabilities to achieve empowerment. This was exemplified in studies where behavior change interventions were delivered via DHTs, as they often overlooked the sociocultural context within which behavior change activities take place, potentially limiting their effectiveness in fostering empowerment [41]. On the other hand, DHTs offering strong social network features enhanced their emotional and social support capabilities [37,51]. The physician-patient relationship, where patients viewed doctors as more knowledgeable and relied on doctors to make all decisions regarding their health, also hindered the attainment of patient empowerment capabilities [41].

#### Environmental Factors

The lack of internet access and Bluetooth connectivity hindered patients’ capabilities to achieve empowerment [47,68]. Additionally, patients were more inclined to use DHTs to achieve valued capabilities if they were free of charge, user-friendly, and safe [41,59].

### Mapping Capabilities to Each DHT as Resources as Reported by Different Studies

**Figure 5:**
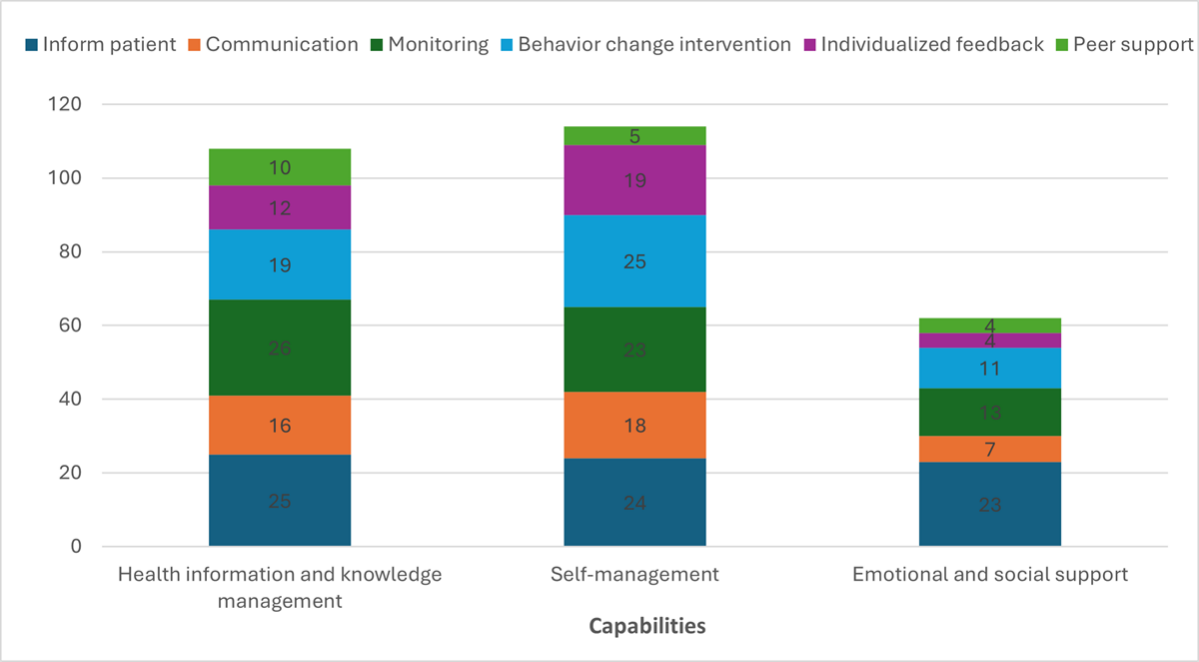
Count of studies by capabilities and digital health technologies as resources.

DHTs as resources enhanced all capabilities, albeit to different extents (Figure 5). This implies the choice of DHT, as a resource depends on the capability we want to enhance. For example, DHTs designed to inform patients about their health and provide monitoring support can enhance patients’ capabilities in both managing health information and engaging in effective self-management. Meanwhile, providing patients with individualized feedback mainly enhances their self-management capability. Very few DHTs as resources in the literature enhanced the emotional and social support capability.

## Discussion

### Principal Findings and Comparison to Previous Work

Drawing on key concepts from the capability approach, this study presents a framework that helps us understand empowerment and its process through DHTs from the patient’s perspective. It identified relevant capabilities valued by patients to achieve empowerment, the different functional components that DHTs offer to support empowerment, and the conversion factors that shape how patients achieve these capabilities. This approach frames empowerment as a broader concept enabling patients to lead the lives they value and improve their health and well-being.

Our findings revealed 9 empowering capabilities when using DHTs that were further grouped into 3 core capabilities, namely, emotional and social support, self-management, and health information and knowledge management. These results align with existing conceptualizations or definitions of patient empowerment that encompass knowledge acquisition, development of skills, and motivation [12,78]. However, we found very few DHTs as resources addressing the emotional and social support capability, which highlights the importance of DHTs to address this capability. The value patients placed on emotional and social support is consistent with the conceptualization and operationalization of health capabilities [79] and relates to certain aspects of Nussbaum’s [31] capability lists of what constitutes a good life, such as bodily health, emotions, practical reason, affiliation, and control over one’s environment.

In line with the capability approach, capabilities not only involve expanding patients’ opportunities and freedom but also respecting their freedom not to act when they choose to [29]. We found that some patients chose not to use DHTs despite the opportunities they provided. For example, patients whose symptoms were well controlled or those experiencing anxiety from information overload did not see any value in using DHTs to become empowered [68]. Empowerment, therefore, is not solely about the expansion of one’s choices but also the freedom to reject certain options when they do not align with personal values or needs.

It is worth noting the interconnectedness of different capabilities, as this can aid in understanding how specific functional components of DHTs lead to these capabilities. For example, providing patients with health-related information enhances their understanding of their health condition and supports them to use this knowledge to improve their health (health information and knowledge management). This, in turn, allows patients to become independent, take necessary steps to improve their symptoms, and contribute more actively to the care relationship based on the available information (self-management). Likewise, patients’ abilities to undertake self-care maintenance activities can be linked to the knowledge they have about their condition and the emotional and social support they get, which increases their confidence in undertaking these activities. This dynamic interaction between patients’ capabilities and the opportunities created by the functional components of DHTs shows an alignment between what patients’ value for empowerment and what DHTs offer to achieve this empowerment.

Using the capability approach, we further identified conversion factors that influence the patients’ capabilities to achieve empowerment through DHTs. These represent the facilitating conditions that are both inherent and external to the individual, such as digital literacy, demographic and socioeconomic status, disease severity, sociocultural norms, doctor-patient relationships, and the cost related to acquiring DHTs. Technology-related factors such as perceived value, ease of use, and safety emerged as important conversion factors. If these barriers can be addressed, patients are more likely to value DHTs as a means to foster their empowerment, ultimately enhancing their health and well-being. These findings align with studies that highlight the barriers and facilitators that influence the uptake of DHTs [24,80,81]. Identifying and overcoming these conversion factors are crucial to enable patients to fully harness the potential of DHTs to achieve their empowering capabilities and integrate these technologies into clinical practice [80,82]. This approach sets our framework apart from prior conceptualizations of patient empowerment, which primarily focus on individuals’ abilities without considering the interplay of internal and external factors that shape their abilities to achieve valued health-related goals [12,78,83].

Surprisingly, very few studies assessed clinical characteristics, such as duration or severity of disease, and the presence of comorbidities [42,49] as useful personal conversion factors that could influence the attainment of capabilities. This gap can be attributed to the fact that studies often include healthier patients [62,70], with limited focus on how their disease state can influence the use of technology. For patients with chronic conditions (eg, those with Parkinson disease), knowing how their motor and cognitive impairments influence their capabilities will provide useful insight into how DHTs can be better adapted to meet their needs.

Our study also revealed that regardless of patients’ demographics or chronic conditions, patients seek similar capabilities and encounter similar conversion factors when using DHTs to achieve empowerment. These findings can be explained by the research context, as all patients were experiencing a chronic condition that necessitated the development of abilities needed to gain control of their lives. The need for patients to develop these abilities has been well documented in studies on the self-management of chronic disease [6,7]. Our results also suggested that patient views on empowerment extend beyond their medical care needs and include capabilities that enable them to pursue activities aligned with their life goals, build supportive relationships, and feel safe while participating in daily activities. Our findings align with current research demonstrating that patients develop capabilities not only to manage their health but also to navigate their personal lives [6,84].

The findings from our review align with and extend existing literature on patient empowerment and digital health [78,85,86]. Previous studies have largely focused on the benefit of DHTs on health outcomes or patient engagement, often without exploring the underlying capabilities that facilitate empowerment [3,22,87]. Our review complements these studies, as it adds information on the individual, interpersonal, structural, and technological aspects of empowerment [12,13,84]. Our use of the capability approach contributes to the field by framing empowerment through the opportunities that patients have when using DHTs to achieve broader health and life goals. This study adds value by demonstrating not only what DHTs offer, and the capabilities patients have to become empowered, but also how their impact can be maximized by addressing conversion factors, ultimately contributing to a more nuanced and actionable understanding of patient empowerment when using DHTs.

### Strengths and Limitations

Our study is the first to apply the capability approach to conceptualize patient empowerment in relation to DHTs. It provides a new theoretical lens that shifts the focus from technology as a mere tool to its role in expanding patients’ substantive freedom and agency in managing their chronic illnesses. By using a theoretical framework, we can capture different empowering capabilities and contribute significantly to the literature on the role of DHTs in fostering empowerment. This is crucial, as the insights gained can directly enhance patient health and management by prioritizing and addressing patient-centered values. This review focused on 5 main chronic conditions; we believe it is broad enough and generalizable to other chronic disease populations with similar needs for empowerment to effectively manage their condition. Another strength of this study is the use of predefined quality criteria to inform our search criteria on different functional components of DHTs. The lack of established methods for reviewing newly developed technologies complicates reviews on DHTs.

Despite the merits of our study, certain limitations must be recognized. First, our search strategy excluded terms associated with patient empowerment in previous research, such as control, patient participation, enablement, or disease-related outcomes. We did not include these terms in our study, since our goal was to learn how patients perceive empowerment, not how researchers, clinicians, or theorists have interpreted it. Including these additional terms would have predetermined which concepts were related to empowerment, potentially shaping our findings based on our own understanding of the term. This exclusion probably resulted in the omission of related papers. However, our study was able to identify some of the disease-related outcomes, like self-efficacy, health literacy, and engagement, as important capabilities for patient empowerment, indicating some alignment with what patients’ value for their well-being.

Another limitation is the absence of a second reviewer at all stages of the review, especially in the qualitative analysis, which could skew our findings based solely on one person’s judgment. To mitigate this bias, we consulted with senior researchers to validate our approach at each step and during the qualitative analysis. Additionally, we followed the best practice guidelines outlined in the PRISMA-ScR checklist to further strengthen our research rigor. The majority of studies were from high-income countries, which represent the needs and states of their health care systems. Additionally, restricting our review to only papers published in English limits our understanding into how digital health interventions and patient empowerment are conceptualized and implemented in different cultural and health care contexts. Consequently, results from this review may not be generalizable to other lower- or middle-income countries and other health care settings.

### Conclusions

This research fills an important gap by using concepts from the capability approach to develop a framework that highlights how patient empowerment occurs through DHTs for chronic disease management. It outlined key capabilities, functional components of DHTs, and conversion factors that contribute to patient empowerment. The capability approach is a valuable framework that helps to understand the personal preferences and variability of patients. Our study serves as a valuable resource for researchers, DHT developers, and policy makers by offering guidance on how to assess and tailor digital health interventions to effectively enhance valued opportunities and consider relevant conversion factors needed to achieve patient empowerment. Future research should strive to highlight and distinguish the effect of different functional components of DHTs on respective capabilities and validate this framework in specific health conditions.

## Appendix

Multimedia Appendix 1 PRISMA-ScR (Preferred Reporting Items for Systematic Reviews and Meta-Analyses Extension for Scoping Reviews) checklist.

Multimedia Appendix 2 Search strategy and summary of eligible studies.

## Abbreviations

DHT: digital health technology
PRISMA-ScR: Preferred Reporting Items for Systematic Reviews and Meta-Analyses extension for Scoping Reviews
