# Supplementary material for: Empowering Capabilities of People With Chronic Conditions Supported by Digital Health Technologies: Scoping Review
Source: J Med Internet Res. 2025 Jun 27;27:e68458. doi: 10.2196/68458 (PMC12254712; doi:10.2196/68458)
Supplement: Multimedia Appendix 2 [file jmir_v27i1e68458_app2.docx]

**Table S1. Search strategy PUBMED.**

| Database | # | Query | Number of articles |
| --- | --- | --- | --- |
| PUBMED | #1 | "patient's"[All Fields] OR "patients"[MeSH Terms] OR "patients"[All Fields] OR "patient"[All Fields] OR "patients's"[All Fields] | 4,417,202 |
|  | #2 | "empowerment"[MeSH Terms] OR "empowerment"[All Fields] OR "empowerments"[All Fields] | 14,394 |
|  | #3 | #1 AND #2 | 5,742 |
|  | #4 | “Digital health” OR “mobile health” OR mhealth OR m-health or “electronic health” or ehealth or e-health or “health information technology” or “virtual care” or telemedicine or “remote consultation” or “telemetry” or telehealth or “wearable device*” or “digital diagnostic*“ or “digital therapeutic*” or “diagnostic software” or “digital software” or “wearable sensor*” or “reminder system*” or “remote monitor*” or telemonitor* or “home monitor*” or “remote care” or “digital technolog*” or “digital medical device*” or “digital health technolog*” or “tele rehabilitation” or “online monitoring” or “remote rehabilitation” or “phone app” or “mobile app” or “mobile application*” or “smartphone app” | 171,361 |
|  | #5 | #2 AND #4  Filters: from January 2013 – April 2024 | 612 |

**Table S2. Description of included studies.**

| **Author (year)** | **Country** | **Research Objectives** | **Study Methods** | **Questionnaire used** | **Target population** | **Disease population** | **Type of technology** | **Description of DHT/intervention** | **Patient-facing category** |
| --- | --- | --- | --- | --- | --- | --- | --- | --- | --- |
| Weymann et al (2015) [73] | Germany | To assess the effectiveness of a web-based tailored IHCA on patient knowledge and empowerment | Quantitative | Health Education Impact Qsn (HEIQ) | Patients | Type 2 diabetes mellitus (T2DM) & chronic low back pain | web-based intervention | Disease related content along with information on its complications; health and behavioral changes and treatment option are delivered via a website in a dialogue format tailored to patient relevant disease characteristics. data collection on patient characteristics occurred during the dialogue and subsequent sections were modified based on response | Care support |
| Skovlund et al (2021) [37] | Denmark | To assess the feasibility, acceptability and perceived benefits and impact of using the digital PRO in routine outpatient care | Mixed method | NA | Patients and healthcare provider (HCP) | T2DM | Web-based | DiaProfil allow patients to complete diabetes related PRO either via phone, tablets or PCs prior to clinic visit. During consultation HCP use the PRO dashboard to plan collaboratively plan patient care. The patient facing component was the PRO questionnaire | Patient monitoring |
| Fairbrother et al (2013) [68] | Scotland | To explore participants views on self management in relation to telemonitoring (TM) | Qualitative | NA | Patients and HCP | Chronic Obstructive Pulmonary Disease (COPD) | TM service | A tablet based device with secure internet where patients can record daily symptoms, medication use and respiratory parameters. Clinical team can review data and contact patients with abnormal results or missing data. it also includes a video link for remote consultation | Patient monitoring |
| Petrocchi et al (2021) [58] | Italy | To assess the feasibility, usefulness and capability of the app to improve PE | Mixed methods | Empowerment scale | Patients | Breast cancer | Mobile app | App with different sections to help patients understand and organize their care journey. Through the app, patient have access to health information and links to useful resources to aid patients at different phases of their cancer journey. The contact and calendar section allows patient to contact members of the care team and keep track of different activities. The document section allows patient to store and share medical files with other doctors and note pad allows them to jot questions for the care team and note taking. | Health & wellness |
| Lustrek et al (2021) [61] | Belgium and Italy | To assess the overall effectiveness of the app, accuracy of patient monitoring features and patient perceptions | Mixed methods | NA | Patients and informal caregiver | Congestive heart failure (CHF) | Mobile app | Combines sensing device and artificial intelligence to encourage self management options for patients. Different sensing devices collect information about patients, which are interpreted by machine Learning algorithm to obtain patient monitoring features. The data is fed to a decision support system which provides recommendation on physical health and psychological support based on the interpreted data | Care support |
| Jaana et al (2018) [66] | Canada | To assess the impact of telemonitoring on patient empowerment and self care and factors associated with thw adoption of TM | Quantitative | PES | Patients | CHF | Tele-monitoring unit | TM system is connected to a cellular or telephone line which transmit daily data on weight, blood pressure and ECG and questions regarding their care to a central TM station. The data is reviewed by a nurse experts who does the follow up with the patient as needed | Patient monitoring |
| Nissen and Lindhardt (2017) [67] | Denmark | To explore patients experience in participating in a 6 month telemedicine intervention | Qualitative | NA | Patients | COPD | Tablet-based | Video consultation plus recording of respiratory parameters 3times a week that is accessed by the respiratory nurse which then follow up the patients | Patient monitoring |
| Marwaa et al (2024) [62] | Denmark | To assess the acceptability of two mobile application to promote cross-sectional, person-centered and empowerment of patients post stroke | Qualitative | NA | Patients and their significant other | Stroke | Mobile app | Studies makes use of two apps: a knowledge-based (Mit Sygehus) for use in the hospital and self-training app to be used at the hospital and rehab centers (Genoptaenjdk). Mit Shngehus contained disease-related information and the rehabilitation process, a journal, appointments, communication with hcp, and information on patient organizations , integration into society and on other rehabilitation centers. The Genoptaenjdk app is integrated with Mit Sygehus and contains generic video recorded exercises. Participants can decide which app to use. Exercises are assigned by the therapist to the personal app of the patient | Health & wellness |
| Pemu et al (2019) [38] | USA | To understand the impact of ehealthystrides on self management behavior and health outcomes | Mixed method | DES | Patients | T2DM | Web-based | e-HealthyStrides is a health coach facilitated intervention. Data from portable devices and clinical data from electronic health records or patient report are connected directly to the platform. Other features include self-directed learning, which is prompted using diabetes curriculum and links to access uploaded self monitored data. Each patient is assigned a health coach, which provide them with resources for tailored behavioural goal setting. Patients also had access to online discussion forum and remote group discussion | Patient monitoring |
| Alharbey and Chatterjee (2019) [50] | USA | To assess the role of technology in improving patient's understanding of COPD and increase behavioral intention towards self-care | Mixed method | NA | Patients | COPD | Mobile app | Consists of an education module to increase patient understanding of disease and risk reduction . The monitoring module allowed patient to self monitor their symptoms and vital signs based on data collected from medical devices and questionnaire. The monitoring module also includes a dashboard that helps caregivers and hcp to intervene when needed. | Patient monitoring |
| Calvo-López et al (2023) [65] | Spain | to develop and evaluate the safety and impact of a home-based cardiac rehabilitation program on patients functional capacity, lifestyle and quality of life | Quantitative | NA | Patients | Ischaemic heart disease | Web-based | Consists regular physical training session and educational session on lifestyle habits, adherence and education. The sessions were conducted via web-based platform under the supervision of a physiotherapist, cardiologist and/or specialized nurse. | Patient monitoring |
| Van den Berg et al (2015) [56] | Netherlands | To assess the effect of ehealth intervention in reducing distress and improving empowerment | Quantitative | Cancer empowerment questionnaire (CEQ) | Patients | Breast cancer survivors | Internet-based | A web-based self-help program delivered during a fixed 16 week modular program without therapist contact. Intervention components include information, assignments (tasks or homework), assessment (self test with automated feedback) and video clips. Materials are released weekly followed by email reminders | Health & wellness |
| Ofili et al (2018) [39] | USA | To assess the acceptability of H360x, efficacy for diabetes self management behaviour change and determine factors for use of technology | Mixed method | DES | Patients | T2DM | Mobile app | An interactive internet-based, patient driven, diabetes self management support and social networking program. *Previously known as ehealth strides.* It consists of an integrated diabetes curriculum and options for uploading and viewing of self monitored clinical parameters in the form of graphics and color coded display. Together with health coach, patients are expected to develop, track and achieve identified self-management goals. Also, there is a discussion forum that allows communication between patients, health coaches and hcp | Patient monitoring |
| Xi Wu et al (2022) [72] | Singapore | To develop a Community-Based e-Health Program (CeHP) for older adults with chronic diseases and conduct a pilot evaluation | Quantitative | PES | Patients | HTN, DM or hyperlipidemia | Mobile app | The ehealth program consists of a face to face and ehealth sessions delivered via an app. The app consist of health education topics, symptoms monitioring function and an alert function for missing data and troublesome readings | Patient monitoring |
| Schmalz et al (2020) [57] | Germany, Finland and switzerland | To assess patients and HCP adoption of the DPMM tool and impact on clinical care | Mixed method | NA | Patients and HCP | Lung Cancer | smartphones, tablets and computers | Technology consists symptoms tracker, communication between patients and hcp, and disease and treatment related educational materials symptoms overview. It also included a symptom overview and alerts for healthcare providers (HCPs) | Patient monitoring |
| Lim et al (2021) [40] | Singapore | to assess the effectiveness of smartphone app based intervention on weight and metabolic outcomes in comparison to usual care | Quantitative | NA | Patients | T2DM | smartphone-based | Participants are provider with app to track their weight, diet and physical activity and to communicate with dietitians along with educational videos. The dietitians regularly reviewed goals with the patients, provided individualized feedback and used motivational techniques to guide lifestyle changes | Patient monitoring |
| Denig et al (2014) [52] | Netherlands | To assess the effect of the decision aid compared with usual care on patient empowerment for setting and achieving goals | Quantitative | DES - I,II,III | Patients | T2DM | Software | Software is linked to the EMR to retrieve patient clinical data and offer personalized information on treatment goals and option to patients | Care support |
| Orozco-Beltrán et al (2022) [41] | Spain | To assess the effectiveness of DeMPower app on metabolic outcomes in people with poorly controlled diabetes | Quantitative | NA | Patients | T2DM | Mobile app | The app is receives data from other connected devices used by patients to periodically measure their glucose, blood pressure, weight and physcial activity. The data is sent to the healthcare team that reviews it, answer patients questions and contact them if needed | Patient monitoring |
| Isernia et al (2020) [70] | Italy | To investigate the efficiency of a Tele-rehabilitation program (HEAD) in terms of adherence and usability, as well as the its effect on motor, cognition and quality of life outcomes among patients with chronic conditions | Quantitative | NA | Patients | Parkinson's Disease | Virtual platform + sensor | A virtual platform that allows for communication between the clinic and the patient's home where game-based rehabilitation activities to improve motor and cognitive function are administered to patients to enable them work towards goal-directed movements in a virtual reality. The physiotherapist and psychologist define the content for each participant prior to each session and the subsequent sessions are adapted based on patient's need and level of disability. A PC, internet and motor capture devices were needed to run the program and was accessible with login credentials. Rehabilitation activities were provided in short video clips | Patient monitoring |
| Miyamoto et al (2019) [42] | USA | To explores the impact of mobile health (mHealth) technology and nurse health coaching on views of diabetes self-management | Qualitative | NA | Patients | TDM | wearable sensor and mobile app | Intervention combines nurse coaching via phone with activity tracking watch and nutrition tracking app. The data was integrated into electronic health records to track daily activity and health behaviour decision by health coaches and healthcare providers | Patient monitoring |
| Schmaderer et al (2021) [63] | USA | To explore the experience of using a self-management mHealth intervention in individuals with heart failure to inform a future mHealth intervention study. | Qualitative | NA | Patients | Heart failure | Mobile app | app to report medications and body weight daily with/without reminders, educational tips and virtual visit by a nurse practitioner or community health workers | Patient monitoring |
| Visser et al (2018) [54] | Netherlands | To assess the effect of my-GMC compared to usual care on psychological distress and patient empowerment | Quantitative | CEQ | Patients | Breast cancer | Mobile app | It is a blended care intervention which combines face to face group medical consultation with an online app. the app is a tablet based online app including several existing apps and connected via a shared icloud account for each group. Patients participated in online support group sessions in the presence of a hcp to address their needs. It also included short video interviews from survivors and documents on information about survivorship. | Health & wellness |
| Jane li et al (2021) [74] | Australia | To understand users’ acceptance of home telemonitoring, their perceived impact, and the implementation challenges in different healthcare organizations | Mixed method | NA | Patients and HCP | COPD, CAD, hypertensive diseases, CHF and diabetes | Home tele-monitoring unit | a home telemonitoring unit where patients report measures required for their care plans at scheduled intervals and a web-based clinical portal where clinicians can review patient data | Patient monitoring |
| Pai et al (2013) [60] |  | To understand patient-s experience with and gather feedback from patients regarding the use of personalize health records | Quantitative | NA | Patients | Prostate cancer | web-based PHR | Patients are given access to their up to date medical record via a portal with access to tools to support them throughout their journey such as messaging, PSA monitoring, decision support tools and presentations | Health & wellness |
| Fu et al (2016) [55] | USA | To describe the development and test of the TOLF (The Optimal Lymph Flow health IT system) | Mixed method | NA | Patients | Breast cancer survivors | Internet-based and mobile based | Web-and-mobile-based educational and behavioral mHealth interventions with instructions on self care exercises to promote lymph flow and improve limb mobility. Website content include information on the disease, self care, daily exercises and ask experts. | Health & wellness |
| Lamprinos et al (2016) [43] | Germany and Turkey | To explore the impact of an ICT-based patient empowerment framework in diabetes self-management | Mixed method | 12 item Scale | Patients and HCP | DM | Mobile and web app | Has a physician and patient component. The patient component involves services for the development and execution of actions to change behaviour according to patient's disease specific needs. It also collects and track data on observations of daily living, physical parameters and physical activity related information. It has a decision support tool that proposed disease management recommendations that is approve by the doctor and presented to the patient | Care support |
| Young et al (2020) [44] | USA | To assess the effectiveness of an intervention combining nurse coaching program with mhealth on self efficacy and management in patients with T2DM | Quantitative | DES-SF | Patients | T2DM | wearable sensor, mobile app and patient portal | It combines nurse-led health coaching techniques with wearable activity, sleep and nutrition trackers that collect and synchronize patient activity generated data into the patient electronic health record. Data is summarized in the form of a dashboard and made available to the health coach and hcp. | Patient monitoring |
| Greenwood et al (2015) [45] | USA | To test the effectiveness of a telehealth remote monitoring intervention with paired glucose testing | Quantitative | DES-SF | Patients | T2DM | Computer-based | A telehealth remote monitoring system, which includes a home table computer connected by internet or 3g network to an online portal. The system is connected to a glucometer via USB cables and has a touch screen for participants to answer daily health session questions as well as brief educational content on self care behavior, and an automated reminder to evaluate glucose data, usage pattern or to revise or continue the following week. A nurse reviews the data and contact patients via virtual visit or short summary of their data with personalized feedback and individualized action care plan | Patient monitoring |
| McGloin et al (2020) [51] | Ireland | To describe the impact on hemoglobin A1c (HbA1c), hypoglycemic events, patient empowerment, diabetes distress, and satisfaction with telemonitoring from the patients’ perspective | Mixed method | DES-SF | Patients and HCP | T2DM | Tele-monitoring unit | Collect and transmits blood glucose reading to nurse who interprets an adjust insulin doses as needed | Patient monitoring |
| McBride et al (2020) [64] | Ireland | To explore patients’ experiences of the usability and feasibility of smartphone apps to support self-management and improve medication adherence in hypertension | Qualitative | NA | Patients | HTN | Mobile app | Smartphone app is a companion to a clinically validated home BP monitor and typically used as a medication management app. It allows patients to self report their BP, stores BP readings, and produce feedback in the form of statistics and interactive charts | Patient monitoring |
| Agarwal et al (2019) [75] | Canada | To explore the feasibility and signals of effectiveness of the Health TAPESTRY-HC-DM approach in supporting the self-management of chronic conditions | Mixed method | Perceived patient empowerment | Patients | T2DM and HTN | web-based app and personal health record | web base tool for data collection and resource provision, which includes a self management module and suggested tip sheets. Community health volunteers used the PHR to conduct regular encounters with patients via home visits, phone calls or secure messaging. Patients complete the modules at home under the guidance of the health connector. Reports are shared and reviewed by the care coordination team | Health & wellness |
| Kehagia et al (2024) [69] | UK | To evaluate the feasibility, acceptability and safety of new home based care pathway for patients with Parkinson Disease | Mixed method | NA | Patients | Parkinson's | Wearable sensor | The wrist worn sensor collects data on motor symptoms and monitor motor function in relation to levodopa. This was combine with other in-person interventions such as group training session and resource packs to support self management. Additionally, Participants were asked to complete hardcopy PROM-based questionnaires. Reports were created form the collected data which is shared with patients and healthcare team. Patients or their caregivers are able to request a healthcare contact when needed. | Patient monitoring |
| Biersteker et al (2021) [71] | Netherlands | To outline the design of the Box and its implementation and use in an outpatient clinic setting | Mixed method | NA | Patients | CVDs - heart failure, Myocardial infarction, atrial fibrillation, and congenital heart diseases | Smartphone compatible devices - consumer graded feedback and not medically graded thus lacking evidence-based accuracy | Consist of disease specific mhealth devices connected via bluetooth to a smartphone and managed through a device dedicated smartphone app. Patients are to measure and report certain parameters (blood pressure, weight scale, Pedometer, ecg, etc) at regular intervals and it was accompanied by videoconsultation with a nurse. The app provided automated feedback based on the readout of the app and flagged abnormal results | Patient monitoring |
| Fundoiano-Hershcovitz et al (2024) [47] | NA | To investigate the efficacy of self monitoring of weight via digital tool on blood glucose levels during the management of diabetes | Quantitative | NA | Patients | T2DM | mobile app and glucose meter | The meter is connected to the phone where blood glucose readings are recorded and weight measures entered regularly. It also included educational content and provides access to personal health information and delivers prompt feedback to users | Care support |
| Koelmeyer et al (2021) [53] | Australia | To gain an in depth understanding of attitudes towards lymphoedema home monitoring using Bioimpedence spectroscopy (BIS) and to explore potential factors associated with acceptance of this approach to self-management for those at-risk of or living with lymphoedema following breast cancer | Qualitative | NA | Patients | Breast cancer related lymphoedema | Portable BIS | Allows individuals to take measurements of body fluid either sitting or standing and the data can be remotely monitored via the internet and stored in a secured cloud | Patient monitoring |
| Hermanns et al (2023) [77] | Germany | To assess the effect of insulin titration using a smartphone app could improve glycemic control | Quantitative | DES | Patients | T2DM | Smartphone app | Has two component, a web portal for physician and the titration app for patients. The web portal is used for participants management where the physician creates a titration scheme which are transferred directly to the patients smartphone and displayed in the app. Additional all entries made by the patients are transferred to the web portal for physician to review and take necessary steps. The patient report daily fasting glucose measurement and the baseline insulin dose, which the app uses to calculate the recommended dose based on the physician setting. Other functionalities of the digital health tool include reminders and notification system | Digital Therapeutics |
| Bollyky et al (2018) [49] | USA | To evaluate the effect of lifestyle coaching in combination with connected glucose device and care support on health outcomes in people with diabetes | Quantitative | DES-SF | Patients | T2DM | Connected devices | Incorporate interventions with different technologies. Participants in the livingo diabetes program received connected two way messaging device that measures blood glucose, stores the data along with other data collected. Based on the reading the device will provide an algorithm driving message about the results along with recommendations. Participants in this group also receive unlimited glucose test strips and access to educators via telephone calls for goal setting, personalized feedback and diabetes education. Participants in the lifestyle coaching arm receive phone calls on goal setting, customized lessons, text messages, meal ratings and activity recommendation. And a connected scale to participants in some intervention groups. | Care support |
| Admiraal et al (2017) [59] | Netherlands | To examine the effectiveness of a web-based tailored psychoeducation program for breast cancer patients | Quantitative | CEO | Patients | Breast cancer | Web-based | Patient completed an online data collection form and received an automatic feedback about their score after which patients received a fully automated tailored psychoeducation for the reported problem | Care support |
| Bond and Worswick (2014) [78] | UK | To evaluate the extend to which the telehealth system supported patient empowerment | Qualitative | NA | Patients and HCP | COPD or heart failure | Home tele-monitoring unit | The telehealth system comprises of monitoring equipment for vital signs and a tablet style computer, which records reading from the monitoring devices and collects other patient data. They result were relayed to the hcp and allow a one way communication from the hcp. | Patient monitoring |
| Hanley et al (2018) [76] | UK | To explore what drove changes to the way tele-monitoring was implemented, compare experience of tele monitoring across the range of long term conditions, and identify what issues, in the experience of the participants, need to be considered when implementing new tele monitoring systems | Qualitative | NA | Patients and HCP | HTN, diabetes, COPD, and heart failure | PC-based or web portal | Reports data from a series of studies on a telemonitoring services. The consistent aspect of the telemonitoring services were that patients collected data on symptoms and vital signs at designated intervals which was sent to hcps via bluetooth, mobile phone or PC who contacted the patients if there were any concern. some of the studies provided educational material | Patient monitoring |
| Morrissey et al (2018) [91] | Ireland | To gain patient perspective on smartphone apps to improve medication adherence in hypertension | Qualitative | NA | Patients | HTN | Smartphone app | It's a self management app for hypertension with two main aspects . 1) medication reminders and 2) home BP monitoring. The home BP monitor collects and sends BP values to the app via Bluetooth and produces a graph of BP measurements | Patient monitoring |
